# Evaluation of the E-Mental Health Mindfulness-Based and Skills-Based “CoPE It” Intervention to Reduce Psychological Distress in Times of COVID-19: Results of a Bicentre Longitudinal Study

**DOI:** 10.3389/fpsyt.2021.768132

**Published:** 2021-11-04

**Authors:** Alexander Bäuerle, Lisa Jahre, Martin Teufel, Christoph Jansen, Venja Musche, Adam Schweda, Madeleine Fink, Hannah Dinse, Benjamin Weismüller, Nora Dörrie, Florian Junne, Johanna Graf, Eva-Maria Skoda

**Affiliations:** ^1^Clinic for Psychosomatic Medicine and Psychotherapy, LVR-University Hospital Essen, University of Duisburg-Essen, Essen, Germany; ^2^Department of Psychosomatic Medicine and Psychotherapy, University Hospital Tübingen, Eberhard Karls University Tübingen, Tübingen, Germany; ^3^Department for Psychosomatic Medicine and Psychotherapy, Otto Von Guericke University Magdeburg, Magdeburg, Germany

**Keywords:** COVID-19, e-mental health, psychological distress, mindfulness, self-efficacy, online intervention, anxiety, depression

## Abstract

**Background:** The SARS-CoV-2 pandemic poses immense challenges for health care systems and population-wide mental health. The e-mental health intervention “CoPE It” has been developed to offer standardized and manualized support to overcome psychological distress caused by the pandemic. The aim of this study was to assess the effectiveness of “CoPE It” in terms of reducing distress (primary outcome), depression and anxiety symptoms, and improving self-efficacy, and mindfulness (secondary outcomes). Furthermore, the intervention's usability, feasibility, and participants' satisfaction with “CoPE It” was evaluated (tertiary outcome). The study protocol has been published previously.

**Methods:** A bicentre longitudinal study was conducted from April 27th 2020 to May 3rd 2021. *N* = 110 participants were included in the analyses. The intervention consisted of four modules featuring different media promoting evidence-based methods of cognitive behavioral therapy and mindfulness-based stress reduction. Difference in psychological distress between baseline (T0) and post-intervention (T1) were analyzed by repeated measure analysis of covariance. Mixed linear models were applied to assess moderating effects. Depressive symptoms, generalized anxiety symptoms, self-efficacy, and mindfulness were compared between baseline (T0) and post-intervention (T1) via *t*-tests. Usability of the “CoPE It” intervention and participants' satisfaction was evaluated by calculation means and frequencies.

**Results:** *Primary outcome*: A significant effect of time on psychological distress at post-intervention (T1) after controlling for age, gender, education, mental illness and attitudes toward online interventions was found. Depressive and anxiety symptoms, and mindfulness were a significant moderators of the relationship between time and psychological distress for consistent wording. *Secondary outcomes:* There was a significant decrease in depressive symptoms and generalized anxiety, and a significant increase in self-efficacy and mindfulness between baseline (T0) and post-intervention (T1). *Tertiary outcomes:* 95.83% of the participants thought the “CoPE It” intervention was easy to use and 87.50% were satisfied with the “CoPE It” intervention in an overall, general sense.

**Conclusion:** The e-mental health “CoPE It” intervention seems to be an effective approach in reducing psychological distress, anxiety and depressive symptoms, and in enhancing self-efficacy and mindfulness during the COVID-19 pandemic. Participants' satisfaction and the program‘s feasibility, and usability were proven to be high.

**Clinical Trial Registration:** www.ClinicalTrials.gov, identifier: DRKS00021301.

## Introduction

In December 2019 the first case of the novel SARS-CoV-2 virus was reported in China (1). Since then, the spread of the virus reached the status of a pandemic, with 212,357,898 people infected and 4,439,843 reported deaths worldwide to this date (2). COVID-19 describes the disease that is caused by infection with the SARS-CoV-2 virus (3). Over the course of time, different approaches to infection control have been implemented by governments all over the world. The government of Germany initiated two lockdowns and varying regional measurements in reaction to three waves of rising infections (4). Many of those restrictions had measurable effects on everyday life and general and psychological health care (5). New virus mutants increased the pace at which COVID-19 could spread (6) and first reports on the long-time effects of infection with COVID-19, a cluster of symptoms called “Long-COVID,” suggest that the pandemic will continue to impact the world in a decisive manner (7).

Several studies investigated the psychological burden that is caused by or goes along with the COVID-19 pandemic (8). Common reactions to the pandemic events are symptoms of anxiety and depression, self-reported distress and sleep disturbance (9). High rates of post-traumatic stress disorder and psychological distress in the general population are reported across different countries (10). These findings were reproduced in data from a large sample of the German population. Since the outbreak of the COVID-19 pandemic, symptoms of depression and anxiety have increased (11, 12). The exact causes of such elevated levels of psychological burden might be manifold. For instance, people who were in quarantine and self-isolated for two weeks showed high levels of anxiety and stress, as well as low sleep quality (13), underlining the negative impact of COVID-19 restrictions on mental health. Also, the pandemic appears to systematically put already vulnerable people at disadvantage: indeed, individuals with preexisting mental health issues are particularly affected by the pandemic, now reaching concerning levels of symptom outcomes (14, 15). COVID-19-related fear represents a pandemic specific stressor that can be found across different countries (16) and highly affects individuals with high-risk diseases (17) and oncological patients (18). In terms of mental health many individuals affected by the pandemic are not attainable in a timely manner. In Lancet Psychiatry, Duan and Zhu urged governments to establish mental health care for those negatively impacted by the COVID-19 pandemic (19). Most people infected with COVID-19 either quarantine at home or are under intensive care in the hospital. Treatment of somatic symptoms of infection with COVID-19 takes priority over treatment of psychological impact. The pandemic places patients in a poor position to access mental health care and psychological treatments were reduced during the early weeks of the pandemic (20). Additionally, waiting times for psychotherapy are long since capacities for outpatient care are limited (21). Overall, since the previous literature convergingly indicates high levels of psychological distress caused by the COVID-19 pandemic, low-threshold mental health support for the general population is required, in order to avert the negative impact of this public health crisis.

The psychological impact of the COVID-19 pandemic and the difficulties in providing psychological support have suggested the need for evidence-based and innovative situation-based approaches to endorse psychological well-being (22, 23). To ensure the acceptance of these new approaches, it is necessary that they are freely accessible, anonymous, and low-threshold (24). E-mental health approaches are versatile instruments that can reach multiple people at the same time. In direct comparison to face-to-face interventions, internet-delivered cognitive behavior therapy shows similar effects in several mental and somatic disorders (25). Nevertheless, establishing e-mental health approaches in health care face several barriers. A study conducted before the outbreak of the pandemic observed ambivalent or negative attitudes toward therapies that were delivered online and the intention to use such therapy approaches was low, even though the participants expected health care improvements if e-mental health approaches were implemented (26). Possible barriers to the use of e-mental health interventions may be concerns about data privacy, lack of quality standards and missing research regarding risks and side effects (27). Personal barriers include–among other factors–time and level of stress (28). For instance, one study assessing the intention to use digital psychodiabetology during the pandemic showed moderate acceptance of such interventions (29).

The ongoing pandemic has already constituted a collective stress test for the implementation of several different eHealth approaches into medical care in order to decrease risk of infection with COVID-19 in face-to-face health care. During the ongoing pandemic, new and digital patient care approaches (e.g., tele-rehabilitation) were rapidly established. These approaches have proven to be highly accepted, satisfying and feasible expansions to medical care (30–35). Regarding e-mental health approaches to face pandemic related distress there is only limited evidence. In fact, there is only one study assessing the efficacy of a therapist-guided online therapy compared to self-help internet-based therapy (36). The goal of this study was to reduce COVID-19-induced anxiety and depression. The study showed that in both groups the levels of anxiety and depression symptoms were reduced, although the reduction in the therapist-guided group was higher. However, it is important to highlight, that this study was not conceptualized to assess the efficacy of a self-guided e-mental health intervention. One different study provides an overview of the intervention protocol of an app-based psychological group intervention as well as preliminary baseline data (37). One study applying qualitative research methods showed overall good acceptance of telehealth to foster mental health. Nevertheless, positive experience of telehealth services were dependent on several factors, including assured support and, comfortable access (38).

These observations demonstrate that we are currently experiencing a shift in establishing new health care approaches, from mandatorily classic face-to-face treatments toward internet-based modalities. However, this development needs to be guided by evidence-based decisions regarding e-mental health interventions in order to quickly adapt to the psychological burden of the general population as well as different patient groups. Only by implementing innovative and easily accessible approaches can the negative impact of the COVID-19 pandemic be reduced at an early stage (39).

The self-guided e-mental health intervention “CoPE It” offers low-threshold support to those who are highly impacted by the psychological strain of the COVID-19 pandemic (40). “CoPE It” is part of the structured clinical approach “Coping with Corona: Extended Psychosomatic care in Essen” (CoPE), which targets psychologically burdened people in Essen, Germany (41). The aim of this study was to assess the effectiveness, usability, and participants' satisfaction with the e-mental health intervention “CoPE It” during the COVID-19 pandemic. The ongoing public health crisis has spotlighted the need for adaption to restrictions of social life and therefore the development of innovative, evidence-based interventions to offer care should be in the focus of clinical research (42). E-mental health interventions like “CoPE It” can bridge the supply gap for higher-threshold interventions like face-to-face therapy.

It was hypothesized that the e-mental health intervention “CoPE It” reduces participants' psychological distress (primary outcome). Secondary hypotheses were that “CoPE It” reduces anxiety and depression symptoms and increases self-efficacy and mindfulness among participants (secondary outcomes). It was further hypothesized that usability of “CoPE It” and satisfaction with the e-mental health intervention would be evaluated positively by participants (tertiary outcomes).

## Materials and Methods

### Procedure and Participants

The previously published study protocol offers an in-depth overview of the methods of the conducted study (43). To investigate the effectiveness of the low-threshold, e-mental health intervention “CoPE It” for psychological burdened individuals during the COVID-19 pandemic, a bicentre longitudinal study was conducted at the University Hospital Essen and University of Tübingen from April 27th 2020 to May 3rd 2021. Potential participants were recruited via the CoPE hotline [see CoPE concept for details (41)], other emergency support hotlines in Germany, via the Health Department Freudenstadt, via the distribution of flyers, from the publicly accessible website, and social media. Eligibility requirements were a good command of the German language, internet access and basic computer skills, and a minimum age of 18 years. Only participants who completed at least the first three modules (out of four) were included into imputation of missing data and statistical analysis. Electronic informed consent was obtained. The study was approved by the Ethics Committees of the University Hospital Essen (20-9243-BO) and University of Tübingen (469/2020BO).

### “CoPE It” Intervention

The self-guided e-mental health intervention “CoPE It” was based on current literature regarding health issues and approaches toward support during the COVID-19 pandemic (44–50). The goal was a reduction of psychological distress by promoting adaptive coping strategies, self-efficacy, daily routines, sleep quality, and activating resources and physical exercises. Evidence-based methods of cognitive behavioral therapy and mindfulness-based stress reduction formed the foundation of “CoPE It” (51–53). The intervention consisted of four modules featuring different media, such as psychoeducational videos, audio-guided mindfulness exercises, and interactive skills training (e.g., planning a daily routine, stress management, activity skills, and individual skills for emotional emergencies). For an overview of the contents of the “CoPE It” intervention, see Table 1. The duration of each module was about 30 min and modules were unlocked in a two-day interval after the completion of the previous module. Details regarding the specific contents of the intervention are provided in the intervention concept and study protocol (40, 43).

**Table 1:** COVID-19 adapted topics, contents, and exercises from the “CoPE It”.

	**Topic**	**Skills Training**	**Mindfulness**
Module 1	• The rationale of the skills and mindfulness training • Rituals and routines	• Planning a daily routine in times of COVID-19 • Activating personal contacts• Enhancing sleep routine	• Mindful breathing
Module 2	• Coping with distress in times of COVID-19 • Stress management	• Stress management model • Encouraging quotes• Self-effective skills	• Mindful experiencing
Module 3	• Individual resources• Resource management strategies	• Activating individual resources in times of COVID-19 • Searching for possible enjoyable activities• Activity skills	• Mindful compassion
Module 4	• Skills box to handle psychological burdens in times of COVID-19	• Individual skills for emotional emergencies • My psychological emergency kit• Reminder skills	• Mindful body awareness

### Measurements

Data for outcome measures were collected via an online assessment tool integrated in the web-based “CoPE It” platform with an approximate completion time of 10–25 min before at baseline (T0) and after completion of the intervention (T1). Primary and secondary outcomes were assessed at both measure points. Attitudes toward e-mental health and demographics were assessed at baseline (T0) and the evaluation of “CoPE It” was assessed at post-intervention (T1). For each of the psychometric instruments, German versions were used. See Table 2 for the assessment schedule.

**Table 2:** Assessment schedule.

**Measures**	**Baseline (T0)**	**Post-intervention (T1)**
**Primary outcome**		
PSQ-20	x	x
**Secondary outcomes**		
PHQ-8	x	x
GAD-7	x	x
GSES	x	x
FMI	x	x
**Evaluation of “CoPE It”**		
APOI	x	
SUS		x
CSQ-I		x
**Sociodemographic and medical data**	x	

*PSQ-20, Perceived Stress Questionnaire-20; PHQ-8, Patient Health Questionnaire Depression Scale; GAD-7, Generalized Anxiety Disorder Scale-7; GSES, General Self-Efficacy Scale; FMI, Freiburg Mindfulness Inventory; APOI, Attitudes Toward Psychological Online Interventions; SUS, System Usability Scale; CSQ-I, Client Satisfaction Questionnaire adapted to Internet-based interventions*.

#### Primary Outcome Measures

The primary outcome is psychological distress at post-intervention (T1), assessed by the German version of the Perceived Stress Questionnaire-20 (PSQ-20) (54).

#### Secondary Outcome Measures

The Patient Health Questionnaire Depression Scale (PHQ-8) and the Generalized Anxiety Disorder Scale-7 (GAD-7) were used to assess depressive and anxiety symptoms (55, 56). To measure self-efficacy, the General Self-Efficacy Scale (GSE) (57) was used. The Freiburg Mindfulness Inventory (FMI) (58) was applied as a measurement of mindfulness.

#### Evaluation of the “CoPE It” E-Mental Health Intervention

The Attitudes Toward Psychological Online Interventions (APOI) instrument (59) was used to assess participants' attitudes toward e-mental health interventions and were considered as covariate. A modified version of the 10-item System Usability Scale (SUS) (60), the Client Satisfaction Questionnaire adapted to internet-based interventions (CSQ-I) (61) were applied for evaluation of usability and participants' satisfaction with the “CoPE It” intervention.

#### Sociodemographic and Medical Data

Self-generated items were used for assessment of participants' sociodemographic information. Demographic data, such as age, gender, marital status, having children, educational level, employment status, and community size were collected. Furthermore, participants were asked about their duration of internet use, prior experience with e-mental health interventions, their possible financial burden due to COVID-19 and if they were either personal affected by a COVID-19 infection or indirectly through infection in their household. Medical data consisted of somatic and mental illness and the use of psychiatric medication.

### Statistical Analyses

Statistical analyses were performed using R (4.0.3). The overall percentage of missing values across the outcome and predictor variables varied between 1.33% and 1.44%, with 329 of 2,640 records (12.46%) incomplete in total. Under the assumption of missing at random, multiple imputation was used for the creation of 100 multiply imputed datasets. Incomplete outcome and predictor variables were imputed using the default settings for predictive mean matching of the ‘mice' package (62). Repeated measure analysis of covariance (ANCOVA) was computed to determine the difference in psychological distress between baseline (T0) and post-intervention (T1). Age, gender, education, mental illness, and attitudes toward psychological online interventions were added as covariates. The variables age and attitudes toward psychological online interventions were included in the model in a standardized form. The variable somatic illness was excluded from the covariates because only 13 participants reported a somatic condition, leaving entire cells empty. Variance inflation factors (VIF) below 1.2 indicated that multicollinearity was not a concern. Partial η^2^ was used as effect size, with values around 0.01, 0.06, and 0.14 considered small, medium-sized, and large effects, respectively (63). Additionally, generalized estimating equations of the original model that actually could include somatic illness were calculated. Mixed linear models with the respective participant as a random intercept were applied to assess moderating effects of age, gender, education, mental illness, and baseline levels of depressive symptoms, generalized anxiety, mindfulness and attitudes toward online interventions on the effect of the intervention on the reduction of psychological distress (primary outcome). All continuous variables were standardized before analysis. There were no outliers, collinearity was low (for all variables: VIF = 1.00) and the error variance turned out to be homoscedastic, as revealed by a Breusch-Pagan-Test. Residuals were normally distributed, except for the model including mindfulness as a moderator. Shapiro-Wilk tests indicated normality of random effects for all models of significant moderating effects. For the secondary outcomes, two-sided paired *t*-test comparing depressive symptoms, generalized anxiety, self-efficacy, and mindfulness between baseline (T0) and post-intervention (T1) were conducted. Due to the sample size, normal distribution was assumed (64). Cohen's *d* was used as effect size, with values around 0.2, 0.5, and 0.8 being considered small, medium, and large effects, respectively (63). To evaluate the “CoPE It” e-mental health intervention, distributions, means and sum scores for SUS and CSQ-I were computed.

## Results

### Study Population

The baseline-data from 440 individuals was collected, of which 114 participants finished module 3 and 4. One hundred thirty-eight participants completed the T0 assessment but did not start the first module. One hundred twenty-eight participants dropped out of the study after the first module and 60 participants dropped out after the second module. The overall dropout rate of this study was 74.09%. Four participants were excluded because they did not fulfill the participation requirements and 110 participants were included into imputation of missing data and data analysis.

Of the 110 participants, 92 (83.6%) were female and the mean age was *M* = 45.09 (*SD* = 14.03). Ninety-three participants (84.5%) had general higher education or entrance qualification for general higher education. Sixty-three participants (57.3%) reported a diagnosis of mental illness in the past. Most participants (*n* = 97, 88.2%) had never used an e-mental health intervention before. For a more detailed sample size description, see Table 3.

**Table 3:** Sociodemographic and medical data.

**Overall**	***n* = 110**
Age [mean (SD)]	45.09 (14.03)
Gender = female (%)	92 (83.6)
Marital status (%)
Single	45 (40.9)
Married/in a relationship	54 (49.1)
Other	11 (10.0)
Children = none (%)	62 (56.4)
Educational level = Higher education (%)	93 (84.5)
Employment (%)	
In education	17 (15.5)
Employment	64 (58.2)
No employment	29 (26.3)
Community size (%)	
100,000 residents	74 (67.3)
<100,000 residents	36 (32.7)
Duration of internet use (%)	
0–1 h	13 (11.8)
1–2 h	22 (20.0)
2–3 h	32 (29.1)
3–4 h	15 (13.6)
More than 4 h	28 (25.5)
Financial burden due to COVID-19 pandemic (%)	
None	73 (66.4)
Low	22 (20.0)
Medium	15 (13.6)
Experience with e-mental health interventions = no (%)	97 (88.2)
COVID-19 (%) = Not affected	104 (94.5)
Somatic illness = none (%)	97 (88.2)
Mental illness = yes (%)	63 (57.3)
Psychiatric medication = no (%)	90 (81.8)

### Primary Outcome Measure: Perceived Stress Questionnaire-20

PSQ-20 scores range between 20 and 80, with higher scores indicating a higher level of psychological distress. PSQ-20-scores were higher at baseline (*M* = 48.26, *SD* = 19.35) than at post-intervention (*M* = 31.95, *SD* = 21.51). An ANCOVA revealed a significant effect of time on psychological distress at post-intervention (T1) after controlling for age, gender, education, mental illness, and attitudes toward online interventions [*F*_(1, 104)_ = 22.41, *p* < 0.001, $\eta_{\text{p}}^{2}$ = 0.18]. Similar results were found in the conducted generalized estimating equations (see Supplementary Material).

### Moderation Analyses

**Figure 1:**
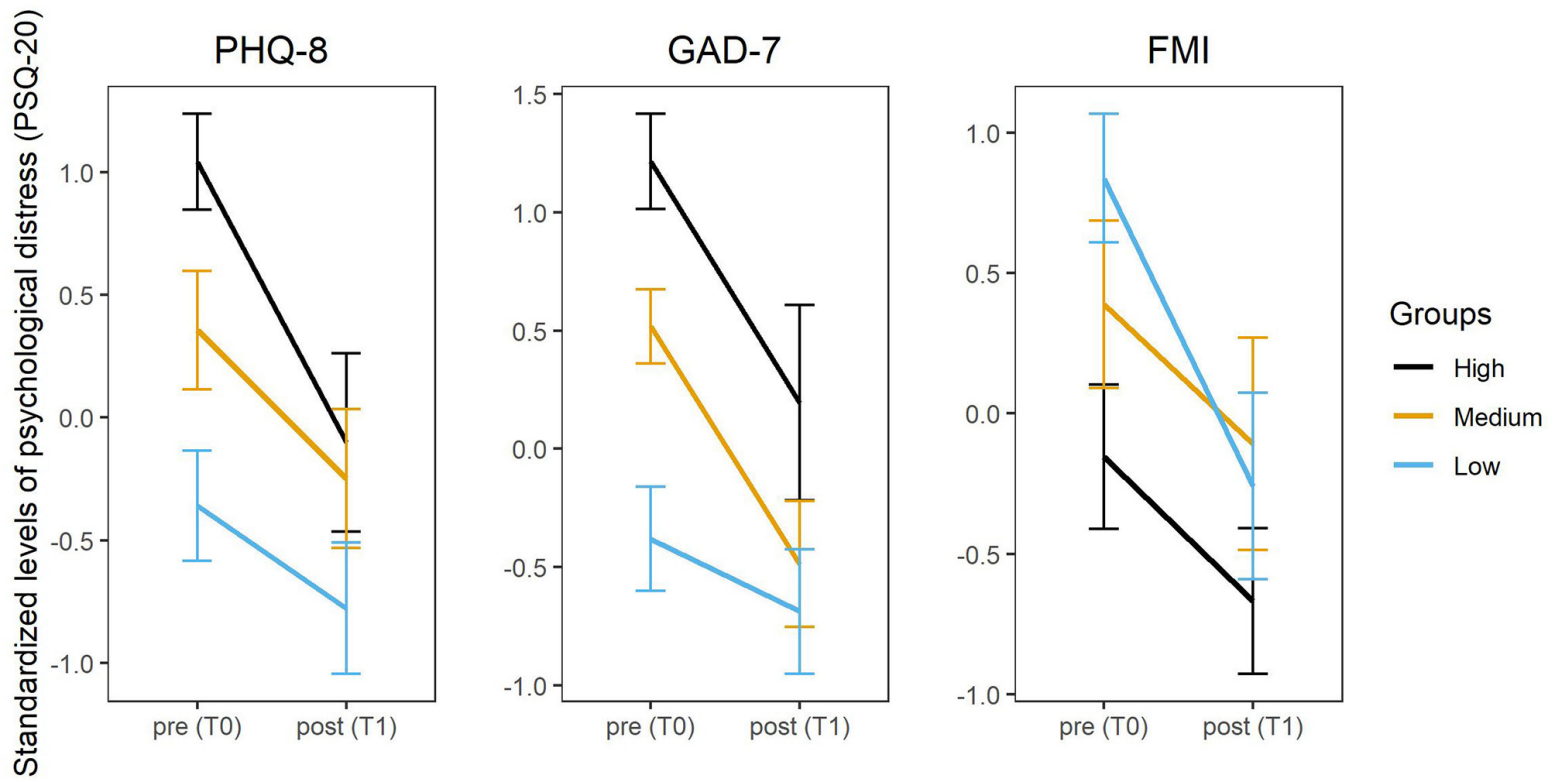
Change of psychological distress (PSQ-20) from baseline (T0) to post-intervention (T1) by group (high, medium, low scores for PHQ-8, GAD-7, and FMI). PHQ-8, Patient Health Questionnaire Depression Scale; GAD-7, Generalized Anxiety Disorder Scale-7; FMI, Freiburg Mindfulness Inventory.

Depressive and anxiety symptoms were a significant moderator of the relationship between time and psychological distress [coefficient PHQ-8^*^timepoint: β = 0.133, *t*(108) = 3.00, *p* = 0.003; coefficient GAD-7^*^timepoint: β = 0.136, *t*(108) = 3.08, *p* = 0.003]. Furthermore, mindfulness significantly moderated the relationship between time and psychological distress (coefficient FMI^*^timepoint: β = −0.145, *t*(108) = −3.39, *p* = 0.001). Estimated marginal effect analyses (see Supplementary Material) suggest that the higher the levels of depressive symptoms and anxiety at T0, and the lower the baseline mindfulness, the larger the effect of the “CoPE It” intervention was. For purposes of illustration of these moderation effects, the sample was divided into tertiles with regard to the respective PHQ-8-, GAD-7-, and FMI-scores and depicted in Figure 1.

### Secondary Outcome Measures: Depressive Symptoms, Generalized Anxiety, Self-Efficacy, and Mindfulness

A measurable and significant change occurred between baseline (T0) and post-intervention (T1) in depressive symptoms [*t*(109) = 6.45, *p* < 0.001, *d* = 0.615], generalized anxiety [*t*(109) = 6.96, *p* < 0.001, *d* = 0.663], self-efficacy [*t*(109) = −4.59, *p* < 0.001, *d* = 0.438], and mindfulness [*t*(109) = −8.98, *p* < 0.001, *d* = 0.856]. The results are depicted in Table 4.

**Table 4:** Results of two-sided paired *t*-tests for secondary outcomes.

	**Baseline (T0)**		**Post-intervention (T1)**				
	***M***	***SD***	***M***	***SD***	***t*(109)**	***p***	***d***
PHQ-8	10.25	5.24	7.21	4.92	6.45	<0.001	0.615
GAD-7	8.79	4.89	5.67	4.25	6.96	<0.001	0.663
GSES	16.78	5.46	18.81	4.12	−8.98	<0.001	0.856
FMI	19.07	7.31	25.68	6.70	−4.59	<0.001	0.438

*N = 110. PHQ-8, Patient Health Questionnaire-8; GAD-7, Generalized Anxiety Disorder Scale-7; GSES, General Self-Efficacy Scale; FMI, Freiburg Mindfulness Inventory*.

### Evaluation of the “CoPE It” E-Mental Health Intervention

SUS scores range between 0 and 100, with higher scores indicating a higher level of usability. For this sample, the SUS score was *M* = 86.18 (*SD* = 10.20, *Mdn* = 86.25). 95.83% of the participants thought that “CoPE It” was easy to use and 87.50% found the various functions in the “CoPE It” intervention to be well-integrated. Further, 88.89% of the participants responded that they would imagine that most people would learn to use this intervention very quickly and 95.83% felt very confident using the system.

CSQ-I scores have a range from 8 to 32, with higher scores indicating higher satisfaction. In this study, the CSQ-I was *M* = 16.99 (*SD* = 5.61, *Mdn* = 18). 94.44% of the participants thought that the “CoPE It” intervention was of high quality. Furthermore, 87.50% of the participants were satisfied with the “CoPE It” intervention in an overall, general sense and 77.78% would come back to such an intervention if they were to seek help again.

## Discussion

The aim of this study was to assess the effectiveness of the e-mental health “CoPE It” intervention, to assess its usability, and participants' satisfaction with the intervention. Participants in the “CoPE It” intervention reported a significant and relevant reduction in psychological distress after using the intervention. The effect was not dependent on age, gender, education, mental illness or attitudes toward online interventions. Higher levels of anxiety and depression at baseline, and lower levels of mindfulness before the intervention were associated with a larger effect of the “CoPE It” intervention. Further, after partaking in the e-mental health “CoPE It” intervention, depressive and anxiety symptoms were lower than before the intervention, while self-efficacy and mindfulness were increased afterwards. Participants evaluated the usability of the intervention as high and were satisfied with the intervention.

Given the results of this study, “CoPE It” seems to have enabled a reduction in psychological distress for individuals during the ongoing COVID-19 pandemic. A large effect was observed. The successful management of distress through evidence-based low-threshold instruments like “CoPE It” provides an innovative approach for prevention of mental health disorders and effective methods to reduce mental burden arising through crises affecting the whole population, such as the COVID-19 pandemic (65, 66). In this study, it was shown that “CoPE It” fulfills these demands and is a helpful intervention in the COVID-19 pandemic to face to increased psychological burden (12). This result is in line with previous research establishing mindfulness-based online interventions and internet-delivered cognitive behavior therapy as effective interventions for stress reduction (25, 53, 67). These new forms of interventions can be applied in everyday clinical practice, therefore lowering the barrier for accessible and low-threshold mental health care.

Even though all participants benefited from the intervention, the positive effect of psychological distress reduction was particularly evident for individuals with higher scores of depressive symptoms and anxiety, as well as participants who reported less mindfulness states at baseline assessment. Similar results were found in an Italian study with female teachers who received a mindfulness training (68). Those with low resilience showed a greater improvement in depression, anxiety, and psychological well-being than those with already high resilience. These observations suggest that “CoPE It” is a valid option to support individuals with a high burden of psychological distress and introduce mindfulness to those who experience less mindfulness in everyday life.

Further, the “CoPE It” intervention reduced symptoms of depression and anxiety. High effect sizes were observed. In accordance with other research, this study provides additional evidence that e-mental health interventions may be able to successfully alleviate the burden of depression and anxiety symptoms (69, 70). This is of particular value since most studies reported an increase in symptoms of depression and anxiety during the pandemic (10–12). The “CoPE It” intervention provides a benefit for those with an already high psychological burden who are in urgent need of support through mental health care.

After participating in the “CoPE It” study, an increase in self-efficacy and mindfulness could be observed. High to moderate effect sizes were found. These skills are important resources for good mental health and dealing with stressful life events (71, 72). Self-efficacy has been found to be a protective factor for nurses working in Wuhan from anxiety related to the COVID-19 pandemic (73). The positive effect of self-efficacy is not limited to health care workers, the general population benefits from self-efficacy as a protective factor against COVID-19 related anxiety, as well (74). High self-efficacy has also been associated with lower psychological distress caused by the COVID-19 pandemic (75). Another study could observe the negative effects of the ongoing pandemic on student's academic self-efficacy (76). Mindfulness has been found to increase well-being, even during the COVID-19 pandemic (77, 78) and was found to comprise a buffering influence on the relationship between fear of COVID-19 and hopelessness (79). Furthermore, there might be a direct impact on the infection risk for COVID-19 since mindfulness is associated with greater engagement in social distancing (80). Enhancing functional coping skills does not only provide protection for the general population but does also mitigate the psychological strain of those with depressive and anxiety symptoms.

Participants in this study reported that they would use the “CoPE It” intervention frequently and almost all participants found the “CoPE It” intervention easy to use and were confident in using the platform. Further, the “CoPE It” intervention was considered helpful for existing problems by a large proportion of participants and participants of this study expressed that it was likely that they would use the intervention again. These findings are in line with research regarding other e-mental health interventions which revealed participants' high satisfaction, usability and acceptance (81, 82). Satisfaction with the intervention was generally good. Regarding usability, responses were also very positive, indicate an user-friendly web-based approach. The results of satisfaction und usability assessment show that the acceptance of “CoPE It” is comparable to other low-threshold interventions.

Global health crises such as the COVID-19 pandemic present a risk for new onset of mental disorders and worsening of existing symptoms such as depression and anxiety. Additionally to these findings, increased psychological distress is a central result of a population-wide strain (74). The e-mental health “CoPE It” intervention addresses these pandemic related strains and offers substantial support to overcome these burdens. Evidence-based e-mental health interventions such as “CoPE It” are urgently needed and are of great value if they can provide relief in times of restrictions even without face to face contact.

### Limitations

The primary limitation is the absence of a control group. Therefore, randomization of study participants could not be performed. Due to non-randomization it could not be ruled out that other factors besides the “CoPE It” intervention may have positively affected the participants. For example, the participants may have adapted to the challenges of the ongoing pandemic over time, thus decreasing the negative effect of pandemic stressors on their mental health. However, this consideration does not explain the increase in mindfulness observed in the study. The results of the e-mental health “CoPE It” intervention are promising, even though it is not yet possible to clearly evaluate its efficiency. A randomized controlled trial to clearly evaluate the effectiveness of the “CoPE It” intervention is needed. However, due to the high psychological distress caused by the pandemic, which affects most people, conducting a randomized controlled trial was ethical not reasonable from our point of view. Because only post-intervention effects were analyzed, no conclusion can be drawn about the long-term effects of the intervention. Further studies with longer follow-up periods are needed to investigate the duration of treatment effects. Moreover, the sample of this study was mostly composed of women with higher education, underlying the need for further studies with a more representative sample of participants. Women seem to be particularly psychologically burdened by the pandemic (8, 9, 12). Since only a few participants were affected by somatic illness or infection of COVID-19, the intervention may have been tested on a group that is less burdened than those in need of e-mental health interventions.

## Conclusion

The e-mental health “CoPE It” intervention seems to be an effective approach in reducing psychological distress, anxiety and depressive symptoms, and enhancing self-efficacy and mindfulness during the COVID-19 pandemic. Participants' satisfaction, and the program‘s feasibility and usability were high. In times of public contact restriction and strain on the health care system, low-threshold e-mental health programs like “CoPE It” could potentially bridge the supply gap of interventions for mentally burdened people.

## Data Availability Statement

The original contributions presented in the study are included in the article/Supplementary Material, further inquiries can be directed to the corresponding author.

## Ethics Statement

The studies involving human participants were reviewed and approved by the Ethics Committees of the Medical Faculty of the University of Duisburg-Essen (20-9243-BO) and the Ethics Committees of the Medical Faculty of the University of Tübingen (469/2020BO). The participants provided their electronic informed consent to participate in this study.

## Author Contributions

AB, E-MS, and MT initiated the study. AB, JG, E-MS, and MT contributed to designing the study and developing the intervention. AB administering the trial and drafted the manuscript with LJ. CJ developed the information technology structure for the study and intervention as well as contributing to preparing the manuscript. Statistical analyses were conducted by LJ, AS, and AB. JG, E-MS, MT, MF, VM, AS, BW, HD, ND, and FJ contributed to the preparation of the manuscript. All authors supported recruitment of the participants and read and approved the final manuscript.

## Funding

We acknowledge support by the Open Access Publication Fund of the University of Duisburg-Essen.

## Conflict of Interest

The authors declare that the research was conducted in the absence of any commercial or financial relationships that could be construed as a potential conflict of interest.

## Publisher's Note

All claims expressed in this article are solely those of the authors and do not necessarily represent those of their affiliated organizations, or those of the publisher, the editors and the reviewers. Any product that may be evaluated in this article, or claim that may be made by its manufacturer, is not guaranteed or endorsed by the publisher.

## Abbreviations

APOI: Attitudes Toward Psychological Online Interventions
CoPE: Coping with Corona: Extended Psychosomatic care in Essen
FMI: Freiburg Mindfulness Inventory
GAD-7: Generalized Anxiety Disorder Scale-7
GSE: General Self-Efficacy Scale
PHQ-8: Patient Health Questionnaire Depression Scale
PSQ-20: Perceived Stress Questionnaire-20
SUS: System Usability Scale
CSQ-I: Client Satisfaction Questionnaire adapted to internet-based interventions.
