# Supplementary material for: Evaluation of the E-Mental Health Mindfulness-Based and Skills-Based “CoPE It” Intervention to Reduce Psychological Distress in Times of COVID-19: Results of a Bicentre Longitudinal Study
Source: Front Psychiatry. 2021 Nov 4;12:768132. doi: 10.3389/fpsyt.2021.768132 (PMC8599585; doi:10.3389/fpsyt.2021.768132)
Supplement: Supplementary file 1 [file Data_Sheet_1.pdf]

Supplemental Material

Evaluation of the e-mental health mindfulness-based and skills-based ‘CoPE It’ intervention to reduce psychological distress in times of COVID-19: Results of a bicentre longitudinal study

Bäuerle et al. (2021)

Regarding 3.2 Primary outcome measure: Perceived stress

Table 2. Results of generalized estimating equations

Effect	Estimate	*SE*	Wald	*p*
(Intercept)	0.505	0.257	3.875	.049
Time: t1	-0.740	0.121	37.605	< .001
Age	-0.053	0.066	0.654	.419
Sex: female	0.238	0.163	2.133	.144
Education: Lower education	0.252	0.171	2.177	.140
Somatic illness: none	-0.228	0.231	0.970	.325
Mental illness: yes	-0.300	0.133	5.120	.024
APOI	-0.010	0.053	0.034	.855

Note. APOI = The Attitudes Towards Psychological Online Interventions instrument.

Regarding 3.3 Analysis of moderation

Estimated marginal means with 95% confidence intervals indicate that higher depressive symptoms and anxiety at baseline, as well as lower mindfulness at baseline result in larger effects at post-intervention (T1). See Figure 1a-c.

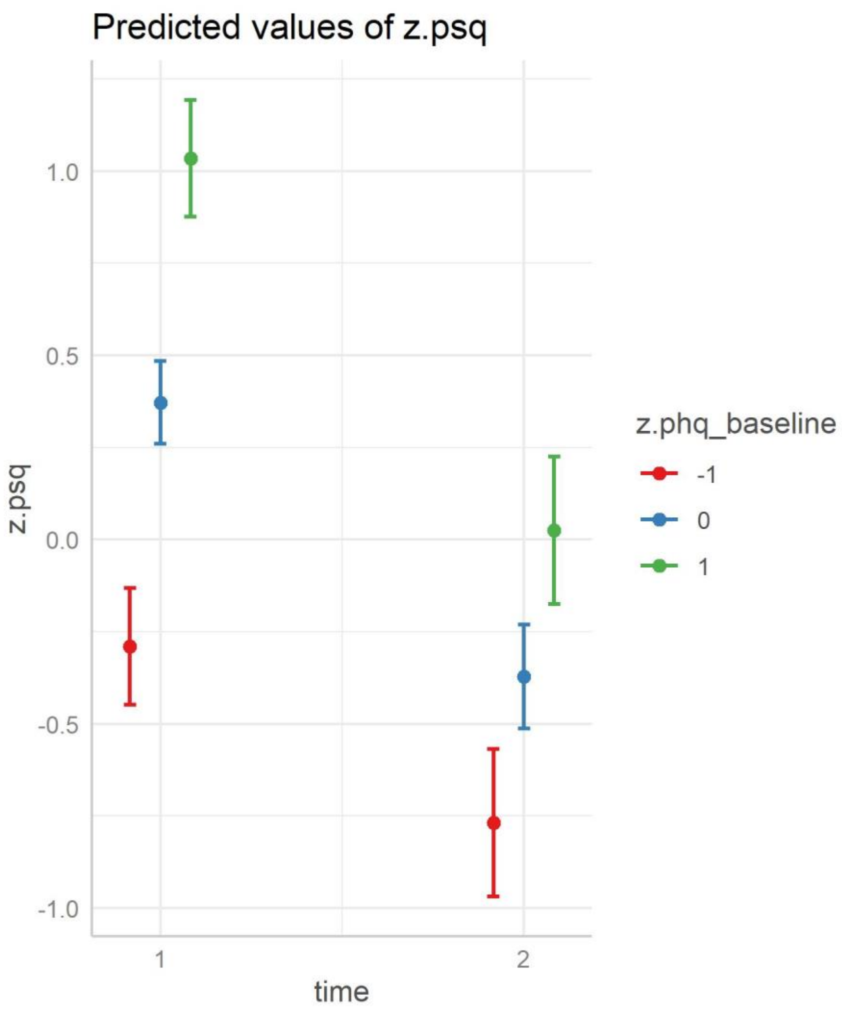

Figure 1a. Predicted values of PSQ-20 (standardized) for PHQ-8 (standardized) at baseline (T0). PSQ-20 = Perceived Stress Questionnaire-20; PHQ-8 = Patient Health Questionnaire Depression Scale.

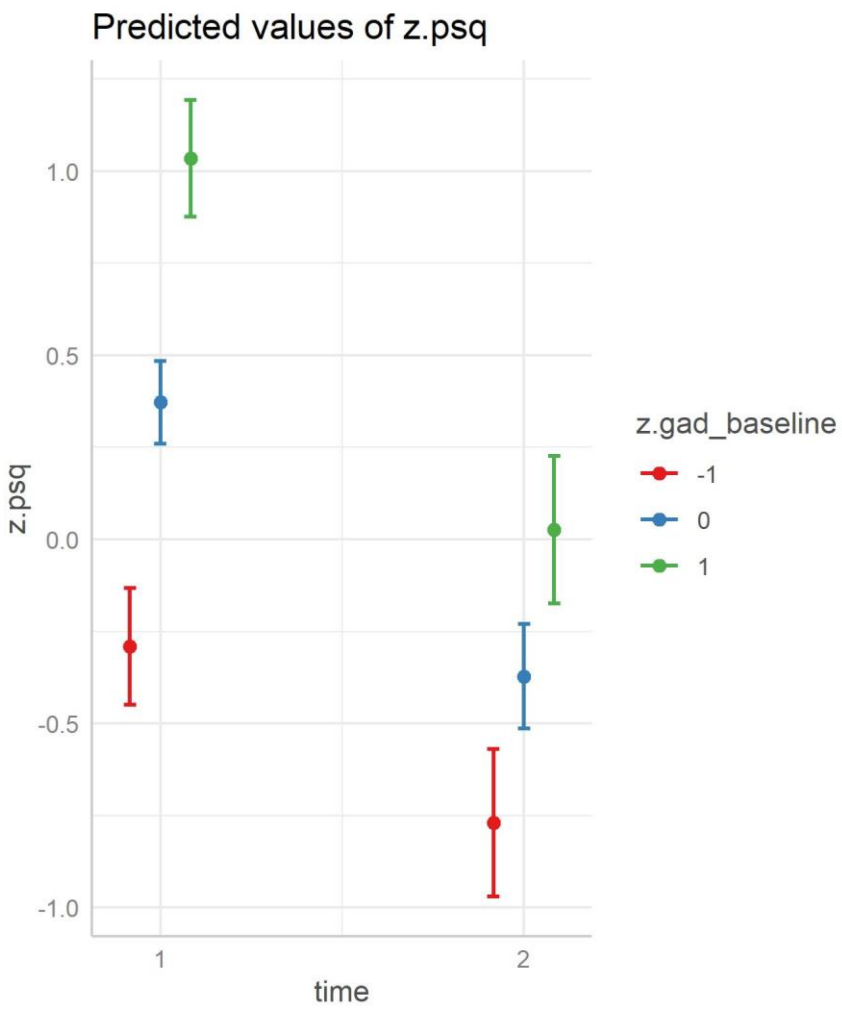

Figure 1b. Predicted values of PSQ-20 (standardized) for GAD-7 (standardized) at baseline (T0). PSQ-20 = Perceived Stress Questionnaire-20; GAD-7 = Generalized Anxiety Disorder Scale-7.

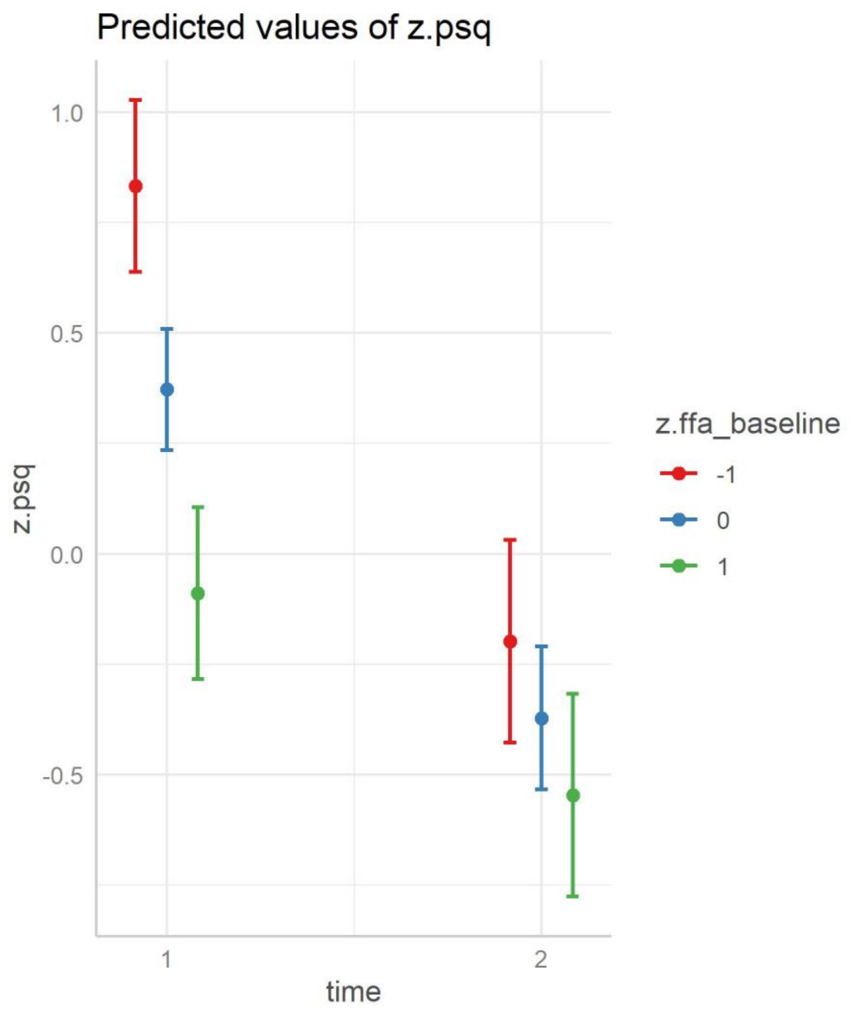

Figure 1c. Predicted values of PSQ-20 (standardized) for FMI (standardized) at baseline (T0). PSQ-20 = Perceived Stress Questionnaire-20; FMI = Freiburg Mindfulness Inventory.
